# Epigenetic regulation of macrophage activation in chronic obstructive pulmonary disease

**DOI:** 10.3389/fimmu.2024.1445372

**Published:** 2024-08-14

**Authors:** Feng Zhang, Yachao Cui, Tiejun Zhang, Wenguang Yin

**Affiliations:** ^1^ State Key Laboratory of Respiratory Disease, National Clinical Research Center for Respiratory Disease, Guangzhou Institute of Respiratory Health, the First Affiliated Hospital of Guangzhou Medical University, Guangzhou Medical University (GMU) - Guangzhou Institutes of Biomedicine and Health (GIBH) Joint School of Life Sciences, Guangzhou Medical University, Guangzhou, Guangdong, China; ^2^ Guangzhou National Laboratory, Guangzhou International Bio Island, Guangzhou, Guangdong, China; ^3^ The Guangdong-Hong Kong-Macau Joint Laboratory for Cell Fate Regulation and Diseases, State Key Laboratory of Respiratory Disease, Guangzhou Medical University Affiliated Qingyuan Hospital, Qingyuan People's Hospital, Guangzhou Medical University, Guangzhou, Guangdong, China

**Keywords:** COPD, macrophage, epigenetic regulation, DNA methylation, histone modifications

## Abstract

Macrophages in the innate immune system play a vital role in various lung diseases such as asthma, chronic obstructive pulmonary disease (COPD), acute lung injury and pulmonary fibrosis. Macrophages involved in the process of immunity need to go through a process of activation, including changes in gene expression and cell metabolism. Epigenetic modifications are key factors of macrophage activation including DNA methylation, histone modification and non-coding RNA regulation. Understanding the role and mechanisms of epigenetic regulation of macrophage activation can provide insights into the function of macrophages in lung diseases and help identification of potential therapeutic targets. This review summarizes the latest progress in the epigenetic changes and regulation of macrophages in their development process and in normal physiological states, and the epigenetic regulation of macrophages in COPD as well as the influence of macrophage activation on COPD development.

## 1 Introduction: COPD and macrophage

Chronic Obstructive Pulmonary Disease (COPD) is the third leading cause of disease death globally. In 2019, COPD accounted for approximately 3.3 million fatalities, imposing a considerable economic burden worldwide (1). COPD patients of China account for around one-fourth of the global COPD population. Notably, China has the highest number of COPD related deaths, with estimated 1.04 million fatalities in 2019 (2). It is estimated that countries that are most severely affected by COPD will be China and the United States from 2020 to 2050. The high prevalence of COPD in China is likely caused by risk factors such as relatively high smoking rates and air pollution. Epidemiological studies indicate that the overall prevalence of COPD is approximately 8.6% in the Chinese population aged 20 years and above. Notably, the number of smokers has reached a staggering 308 million aged 15 years and above, and the smoking rate among adult males has reached an alarming 50% (3).

COPD is characterized by symptoms including dyspnea, cough and sputum production (4). This heterogeneous disease is characterized by persistent respiratory symptoms and airflow limitation due to small airway inflammation and remodeling, mucus obstruction and alveoli disruption (5). Multiple genetic and environmental factors influence COPD development. The most common risk factor for COPD is long-term active or passive exposure to cigarette smoke. Cigarette smoke is a complex aerosol containing numerous toxic and carcinogenic compounds including over 4,700 different chemicals and 1,014 free radicals and oxidants (6). Inhaled cigarette particles are deposited in the respiratory tract based on size, with larger particles mainly locating in the upper airways and smaller ones mostly locating in the alveoli. These particles induce oxidative stress, which recruits inflammatory cells such as macrophages, neutrophils and T lymphocytes to the airways to cause chronic inflammation and exacerbate the inflammatory response (7). Repeated long-term exposure to cigarette smoke significantly heightens the risk for this disease (8).

The primarily aims of clinical treatment of COPD are to alleviate symptoms, improve patient health and enhance life quality. However, interference with the progression of COPD may also cause side effects on this disease. COPD is a heterogeneous disease that may require precise and personalized treatment (9). Identification of new COPD biomarkers is also crucial for establishing diagnostic criteria in the early stage of this disease and predicting treatment outcomes (10, 11).

The lungs are important respiratory organs, which facilitates the exchange of gas between the blood and the environment and clear particulate matter, bacteria, viruses, allergens and other harmful substances (12). The immune system maintains normal physiological function of the lungs through immune responses and also protects them from being damaged by endogenous and exogenous factors. Macrophages, vital myeloid cells within the immune system, are crucial for engulfing and eliminating pathogens and clearing damaged or senescent cells, which is vital for tissue repair (10). Macrophages locate in many tissues, and their differentiation and tissue-specific activation can be regulated by epigenetic control such as DNA methylation, histone modification and changes in chromatin structure. The epigenetic regulation of macrophages is shaped by lineage and tissue-specific transcription factors, stemming from intrinsic myeloid development program and environmental tissue signals. Macrophages adapt to microenvironmental changes by modifying gene expression profiles and epigenetic states (13).

COPD patients always exhibit elevated macrophages, neutrophils, T lymphocytes and innate lymphoid cells in the lungs notable and also an increase in numbers of macrophages in sputum and bronchoalveolar lavage fluid (14). Lung macrophages and neutrophils secrete substantial quantities of tissue-damage enzymes including elastase, metalloproteinase 2 (MMP-2), MMP-9, MMP-12 and cathepsin-S in response to foreign particles and microorganisms stimulation. These enzymes contribute to elastin degradation, leading to destruction of the alveolar wall (15). Continuous exposure to cigarette smoke or bacteria, key triggers for airway diseases such as COPD and asthma, markedly diminishes intracellular antioxidants and induces severe oxidative stress. Macrophages recruit more immune cells, which can exacerbate tissue damage and compromise immune surveillance and phagocytic functions (16).

Macrophage involvement was closely linked to the development of COPD. A clinical study using the M2 marker CD163 examined bronchoalveolar lavage (BAL) from 114 COPD patients, including 72 current smokers and 42 ex-smokers who had quit smoking for an average of 3.5 years. The results showed that ex-smokers had a higher percentage of anti-inflammatory M2 macrophages (83.5%) compared to current smokers (68.0%), indicating that smoking cessation significantly affected macrophage polarization in the lungs (17). Further research comparing COPD patients to healthy individuals found that as COPD severity increased, there was a rise in BAL fluid of unique macrophage groups that were poorly polarized. These included non-M1 (CD40 positive) and non- M2 (CD163 positive) macrophages. These poorly polarized macrophages were pro-inflammatory and had reduced phagocytic abilities. In COPD patients, dual-polarized macrophages had the highest phagocytic index at 77.4%, followed by M1 macrophages at 38%, M2 macrophages at 35.7%, and the least effective were the depolarized macrophages at 11.0% (18). A preclinical study with mice developed a COPD model using CS exposure combined with CES intraperitoneal injections. It found that after modeling, macrophages tended to polarize towards the M2 phenotype and activated the TGF-β/Smad signaling pathway. There was a positive correlation between the M1 to M2 ratio and airway resistance (Raw), and a negative correlation with lung dynamic compliance (Cdyn), peak expiratory flow (PEF), and the ratio of inspiratory to expiratory time (Ti/Te) (19). Studies involving human small airway tissues, bronchoalveolar lavage, and a COPD mouse model showed that macrophages in the airways of smokers with normal lung function and COPD patients primarily shifted towards the M1 phenotype; however, in BAL fluid, they shifted towards the M2 phenotype. In the COPD mouse model, there was an increase in M2 cytokines such as CCL22, IL-4Rα, and IL-13Rα1, while M1 markers like iNOS/NOS2, IL-12, and IP-10 did not increase (20). These macrophage changes mirrored the epigenetic disruptions seen in epithelial cells in COPD (21), leading to a cascade of pro-inflammatory responses and oxidative stress that further damaged the lungs and contributed to disease progression (22).

In summary, macrophage alterations in COPD are significant, playing a crucial role in the onset and progression of the disease. Epigenetic modifications serve as an important mechanism for regulating macrophage phenotype. Therefore, this review will focus on the epigenetic research related to COPD and macrophages, attempting to summarize how these genetic changes influence macrophage function and the progression of COPD.

## 2 Epigenetic change in COPD

Genome-wide association studies (GWAS) in patients have revealed that epigenetic changes also play a role in pulmonary diseases such as COPD, asthma and pulmonary hypertension (23, 24). Epigenetic mediation is primarily achieved through DNA methylation and histone modification without alteration in DNA sequence. Epigenetic modifications can be caused by aging and environmental changes, and these changes can be passed to offspring through mitosis and meiosis (25).

### 2.1 DNA methylation

DNA methylation is an epigenetic regulation mechanism in the mammalian genome, typically occurring at CpG dinucleotide sites and involving the transfer of a methyl group to the C5 position of cytosine to form 5-methylcytosine. The genomic DNA methylation pattern can alter through dynamic processes of methylation and demethylation. The methylation process is facilitated by DNA methyltransferases (DNMTs), where DNMT3a, DNMT3b and DNMT3c establish *de Novo* methylation at unmethylated CpG sites, and DNMT1 primarily maintains methylation during cell division (26). Passive demethylation results from the gradual loss of methylation marks during cell division, whereas active demethylation is facilitated by the Ten-eleven translocation (TET) methyl cytosine dioxygenase family proteins. TET enzymes can iteratively oxidize 5-methylcytosine (5mC) to modify DNA to produce intermediates such as 5-hydroxymethylcytosine (5hmC), 5-formylcytosine (5fC) and 5-carboxylcytosine (5caC). These intermediates are further converted into unmethylated cytosine through base excision repair mediated by thymine-DNA glycosylase (TDG) (27). Therefore, differentiated cells develop stable and unique DNA methylation patterns that regulate tissue-specific gene transcription (28).

COPD is closely associated with cigarette smoke and aging, characterized by respiratory inflammation, mucociliary dysfunction, emphysema and airway fibrosis. Current molecular studies on COPD primarily explore the balance between oxidants and antioxidants, chronic inflammation, apoptosis, the elastase-anti elastase hypothesis and abnormal tissue repair (21). DNA methylation suppresses gene activation. In COPD patients, alterations in DNA methylation levels at pro-inflammatory gene promoters in alveolar epithelial cells and macrophages are pivotal to COPD pathogenesis. These epigenetic modifications vary with age and severity of exposure to cigarette smoke (29). Cigarette smoke extract (CSE) markedly activates the DNA demethylase enzyme TET. TET1/2 are vital in regulating DNA methylation of genes such as NF-κB, STAT3, IKK and NIK, leading to changes in the production of cytokines and chemokines in A549 cells. Exposure to cigarette smoke notably reduces DNA methylation at the CpG promoter regions of inflammation-related genes such as IKK and NF-κB in the NF-κB pathway (30). TET enzymes facilitate stepwise DNA demethylation (31), whereby TET1 decreases methylation at CpG promoter sites of STAT3, JAK1 and PIAS3, consequently elevates production of inflammatory cytokines and chemokines such as interleukin-8 (IL-8) which attracts neutrophils (32), and monocyte chemoattractant protein 1 (MCP-1) which attracts monocytes (33), and chemokine ligand 5 (CCL5) which attracts T cells (34).

### 2.2 Histone modifications

Histone modifications include acetylation, methylation, phosphorylation, lactylation and ubiquitination. Post-translational modifications (PTMs) of histones represent a critical form of epigenetic modification that can induce changes in chromatin structure and gene expression (35). In eukaryotes, genomic DNA is organized into chromatin including the structural unit known as the nucleosome, housed within the cell nucleus. The nucleosome consists of a histone octamer, featured as one [H3-H4]2 tetramer and two [H2A-H2B] dimers, and is encircled by 145 to 147 base pairs of DNA (36). Within the nucleosome, the eight terminal tails of the histone pairs extend from the DNA, which permits post-translational modifications on their side chains by epigenetic regulation proteins (37). Lysine acetylation, a reversible form of post-translational modification, involves addition or removal of acetyl groups from histones and non-histone proteins by lysine acetyltransferases and deacetylases. Histone acetylation and deacetylation regulate biological processes such as transcription, translation, differentiation, protein folding and degradation (38). Acetyltransferases CBP and p300 are multifunctional transcriptional co-activators. CBP and p300 target thousands of acetylation sites that can rapidly cycle through acetylation and deacetylation within 30 minutes, highlighting the dynamic nature of these modifications. Environmental, metabolic and molecular signals can regulate the activity of lysine acetyltransferases and deacetylases (39).

Chronic smoking is one of main cause of COPD, and also leads to systemic effects including the critical complication of muscle atrophy (40). Abnormal activity of histone deacetylases (HDACs) such as HDAC1 is associated with muscle atrophy and contraction dysfunction. HDAC1 contributes to muscle atrophy by enhancing expression of FOXO transcription factor-dependent genes. These genes are involved in the ubiquitin-proteasome pathway (TRIM63 and FBXO32), autophagy-related process (LC3) and protein synthesis process (4EBP). HDAC inhibitors Trichostatin A (TSA) can alleviate skeletal muscle atrophy, mitigate disease progression from degenerative changes at the neuromuscular junction, and improve survival rates in animal models. These findings suggest that HDAC inhibitors may be therapeutic candidates for treatment of COPD-associated skeletal muscle atrophy (41).

### 2.3 m6A modification

N6-methyladenosine (m6A) is the most common, abundant and reversible post-transcriptional modification in eukaryotic cells. M6A affects pre-mRNA processing, transport, translation efficiency and spliceosome splicing sites, and plays a vital role in post-transcriptional gene regulation (42). The m6A methylation process is mainly controlled by methyltransferases METTL3 and METTL14, which catalyze this modification at adenosine residues in mRNA, the most frequent type of mRNA modification (43). The demethylation process of m6A is regulated by FTO and ALKBH5 (44). Variations in RNA modification levels under different conditions can dictate mRNA destiny and regulate physiological and developmental pathways (45).

In COPD patients, Epithelial-Mesenchymal Transition (EMT) is thought to contribute to airway remodeling. Smoking can activate EMT in airway epithelial cells by up-regulating Mettl3 expression to reduce SOCS3 protein levels through m6A methylation and enhance IL-6/STAT3/SNAI1 signaling. IL-6/STAT3/SOCS3 signaling is crucial in regulating inflammation and EMT (46). COPD is also closely associated with PM2.5 pollution. The increased apoptosis of endothelial cells and reduced capacity of angiogenesis in COPD patients indicate that lung microvascular endothelial cell damage contributes to pathogenesis of this disease. PM2.5 affects cellular apoptosis, cell permeability and angiogenesis via Mettl16-mediated m6A modification, which promotes pulmonary vascular damage in COPD (47).

## 3 Epigenetic regulation mechanism of macrophage development and physiological state

Macrophages are one of the earliest immune cell types that appear in human embryonic development, capable of engulfing dead cells, cellular debris, immune complexes and bacteria. Macrophages produce growth factors and signaling molecules, vital for defending against invading microbes and facilitating organ development. These molecules also regulate angiogenesis, influence metabolism, mediate tissue repair, and oversee the maturation of embryonic tissues, thereby maintaining body homeostasis (48). Macrophage diversity arises from various developmental trajectories, transcriptional programs and life cycles. Macrophages derive from two main pathways: tissue-resident macrophages which develop from embryonic yolk sac progenitors and fetal monocytes, and circulating macrophages that differentiate from bone marrow monocytes (49, 50). Tissue-resident macrophages preserve tissue homeostasis and mediate the suppression and resolution of inflammation. Conversely, monocyte-derived macrophages are recruited to sites of inflammation upon its induction, and play an important role in host defense against pathogens, foreign antigens and tissue damage (51).

### 3.1 Tissue-resident macrophages

In the early stages of embryonic development, erythromyeloid progenitors (EMPs) in the yolk sac are capable of generating primitive yolk sac macrophages before hematopoietic stem cell formation. These macrophages are the exclusive source of certain tissue-resident macrophages such as microglia. Subsequently, macrophages migrate to the fetal liver and transform into fetal monocytes, which proliferate and evolve into various types of tissue-specific macrophages. Notably, these tissue-resident macrophages possess significant self-maintenance and regeneration capabilities (52). During organ and tissue development, tissue-resident macrophages integrate with specific cell types and form a part of the tissue structure. For example, microglia integrate with neurons and form a part of the neural tissue structure in the brain, while adipose-associated macrophages integrate with adipocytes in white adipose tissue and impact the regulation of fat storage. Osteoclasts integrate with osteoblasts in bone tissue, and facilitates the remodeling and maintenance of the skeletal system (53). Compared to macrophages derived from hematopoietic stem cells and circulating monocytes, tissue-resident macrophages display distinct genetic, developmental and functional characteristics (54, 55).

Tissue-resident macrophages are instrumental in tissue development, and sensing and integrating local and systemic signals to support specialized cells. These macrophages are responsible for secreting essential growth factors, enhancing nutrient circulation and facilitating waste clearance. Tissue-resident macrophages are pivotal in organ development and tissue regeneration post-injury, contributing to tissue metabolism and protecting against infectious diseases (53). For example, alveolar macrophages secrete anti-inflammatory substances in the resting state. PPAR-γ regulates antigen presentation to the adaptive immune system, thereby mediating the process of inflammatory activation. All known endogenous ligands for PPAR-γ are derivatives of fatty acids. The macrophage-specific knockout of enzymes that convert esterified fatty acids into free fatty acids results in severe pulmonary inflammation. In alveolar macrophages, PPAR-γ is pivotal in curtailing excessive and potentially harmful inflammation in the lungs (56).

Tissue-resident macrophages display unique gene expression profiles, such as high expression of Sall1 in microglia, Clec4f in Kupffer cells, Tgfb2 in peritoneal macrophages and Car4 in pulmonary macrophages. In similar microenvironments, different types of macrophages exhibit a similar cluster of highly expressed genes. For example, Kupffer cells and splenic macrophages actively participate in the renewal of erythrocytes, with gene expression primarily related to heme and porphyrin metabolism. Genes enriched in small intestine and large intestine macrophages may reflect their response to bacterial and antigen processing (57). Influenced by their microenvironments, tissue-resident macrophages develop a specific enhancer landscape. The combination of tissue-specific and lineage-specific transcription factors establishes a regulatory network that controls their chromatin specialization (58).

### 3.2 Circulating macrophages

In adults, hematopoietic stem cells in the bone marrow serve as another major source of macrophages as evidenced by bone marrow transplant patients. After myeloablative therapy, transplanted hematopoietic stem cells replenish all circulating blood cell types, including erythrocytes, granulocytes, monocytes and lymphoid cells (59). Intestinal macrophages originally derived from yolk sac or fetal liver monocytes are present in the neonatal intestinal mucosa. These macrophages are gradually replaced by classical Ly6C^hi^ monocytes, originating from the bone marrow during newborn mice growing into adult mice. These monocytes are influenced by the chemokine receptor CCR2 and gut microbiota prior to weaning and gradually migrate to the intestinal mucosa and differentiate into mature anti-inflammatory macrophages (60). Once classical Ly6C^hi^ monocytes reach the resident tissue, these monocytes undergo differentiation that is regulated by tissue-specific molecular cues. The monocytes can function to maintain tissue integrity, regulate metabolism and protect against infectious diseases (61, 62).

**Figure 1:**
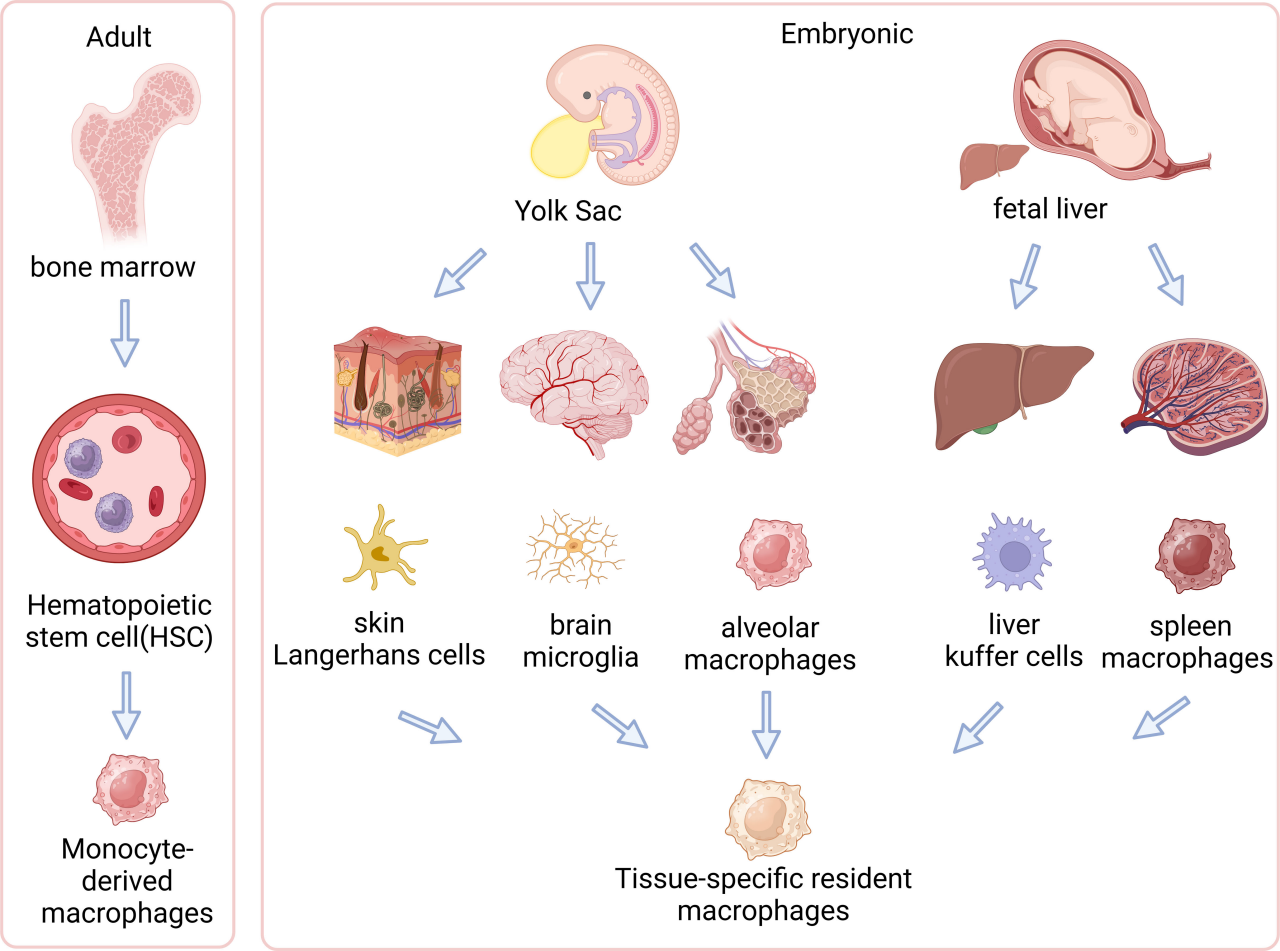
Origins of tissue-resident and circulating macrophages. The left side of the illustration shows adult-derived macrophages, originating from hematopoietic stem cells (HSCs) in the bone marrow, which can differentiate into tissue-resident macrophages within specific tissues or exist as circulating macrophages in the bloodstream. The right side demonstrates embryonic origins, where primitive yolk sac macrophages migrate into the fetal liver and transform into fetal monocytes. These fetal monocytes proliferate and differentiate into various types of tissue-resident macrophages in embryonic tissues, such as microglia in the brain, Kupffer cells in the liver, and alveolar macrophages in the lungs. This diagram illustrates distinct developmental pathways of macrophages in embryonic and adult stages and their localizations in tissues. (Created with BioRender.com).

The origins and evolution of macrophages involve a transition from embryonic yolk sac and fetal liver to adult hematopoietic stem cells. These cells are vital in specific tissues within the body. **Figure 1** illustrates how these macrophages differentiate and adapt across various developmental stages and environments (63–66) (**Figure 1**).

### 3.3 M1/M2 macrophages

Macrophages can be classified into M1 and M2 phenotypes. Macrophages can polarize towards the M1 phenotype by Th1 cytokines (such as IFN-γ and TNF-α) or lipopolysaccharide (LPS) induction. M1 macrophages produce high levels of pro-inflammatory cytokines, such as IL-1β, TNF and IL-12, and highly express MHC-II with strong bactericidal ability. M1 macrophages primarily promote a Th1-type inflammatory response. M1 macrophages inhibit the proliferation of surrounding cells and cause tissue damage, being termed “classically activated macrophages” (67). Th2 cytokines (such as IL-4, IL-10 and IL-13) and some TLR ligands promote macrophages polarization towards the M2 phenotype. M2 macrophages secrete anti-inflammatory cytokines, promote immune regulation through debris clearance, and participate in tissue repair and remodeling. M2 macrophages are termed as “alternatively activated macrophages”. Based on the expression profile, M2 macrophages can be further subdivided into M2a (anti-inflammatory, maintaining tissue homeostasis), M2b (Th2 activation, immune regulation), M2c (phagocytosis of apoptotic cells) and M2d (angiogenesis and tumorigenesis) (68).

In inflammatory environments, granulocyte-macrophage colony-stimulating factor (GM-CSF) and macrophage colony-stimulating factor (CSF-1, also known as M-CSF) play pivotal roles in regulation of macrophage lineage numbers and function (69). These CSFs are crucial for maintaining macrophage homeostasis and development, and mediating physiological and pathological responses. Specifically, M-CSF sustains its expression under non-inflammatory conditions, while GM-CSF is produced predominantly by inflammatory or damaged cells in pathological states (70). *In vitro*, M-CSF regulates the quantity of macrophages and monocytes from various tissue origins without altering their activation status. Monocytes treated with M-CSF differentiate into monocyte-derived macrophages (MDMs), which is commonly used as a model for studying tissue macrophage differentiation (71). Conversely, GM-CSF activates monocytes/macrophages and directs their differentiation into forms involved in immune responses. Monocytes treated with GM-CSF are often used in studies on the development and function of dendritic cells (72). Monocytes treated with GM-CSF exhibit pro-inflammatory M1 macrophage traits (73), while those treated with M-CSF display traits of anti-inflammatory M2 macrophages (74). Additionally, under GM-CSF induction, the transcription factor interferon regulatory factor (IRF5) is instrumental in polarizing human monocytes to M1 macrophages. High IRF5 expression, a characteristic of M1 macrophages, activates expression of genes such as interleukin-12 subunit p40 (IL-12 p40), IL-12 p35 and IL-23 p19, and suppresses IL-10, thereby enhancing the inflammatory response (75).

**Figure 2:**
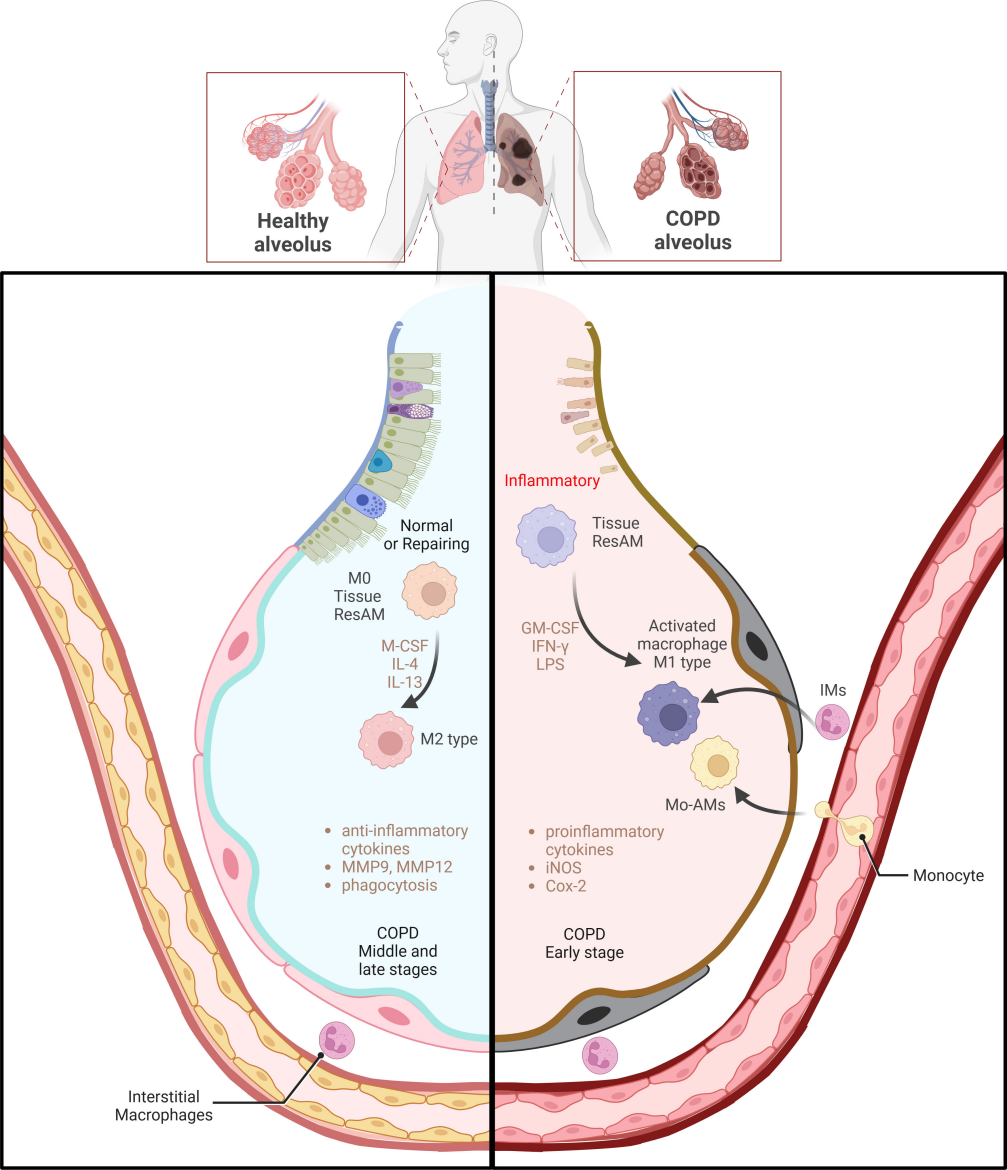
Macrophage polarization and functional regulation in COPD. The left side of the image shows macrophages in healthy alveoli primarily in the M2 state involved in tissue repair and anti-inflammatory processes, which can be regulated by factors such as M-CSF, IL-4 and IL-13, The right side depicts the activation of M1 type macrophages in the alveoli in early stages of COPD. These M1 type macrophages produce high levels of pro-inflammatory cytokines under the influence of GM-CSF, IFN-γ, and LPS, iNOS and COX-2, which enhances the inflammatory response. M0 macrophages, a basal form, can polarize towards M1 or M2 phenotypes under environmental influences. The transition of macrophages from pro-inflammatory to tissue repair regulation during the middle and late stages of COPD emphasizes a shift in macrophage phenotypes during COPD progression and their critical role in disease progression. (Adapted from “Acute Respiratory Distress Syndrome (ARDS)”, by BioRender.com).

In COPD, macrophages play a pivotal pathophysiological role, with their M1 and M2 phenotypes exerting distinct functions. M1 and M2 macrophages modulate inflammatory responses in divergent mannners (76). During infections or smoking-related lung damage, M1 macrophages contribute to a Th1-type inflammatory environment. M1 macrophages secrete high levels of pro-inflammatory cytokines, including IFN-γ and TNF-α. This can exacerbate inflammation and tissue damage surrounding the cells. M2 macrophages are predominantly active in non-inflammatory states and are involved in tissue repair and remodeling. M2 macrophages maintain tissue homeostasis by secreting anti-inflammatory cytokines and clearing cellular debris (10) (**Figure 2**). The composition of macrophages varies significantly among COPD patients, smokers and non-smokers. In healthy non-smokers, lung macrophages are typically not polarized into M1 or M2 phenotypes. However, as COPD severity and smoking increase, the percentages of M1 and M2 macrophages also gradually rise. M1 percentages increase from 26% to 84%, and M2 percentages from 7% to 78%. In patients with severe COPD, M1 and M2 macrophages have been observed to co-localize within the same alveolar macrophages (AMs). This indicates that as the severity of the disease increases, macrophages with M1 and M2 characteristics can coexist, rather than being mutually exclusive (77). In another clinical study on the classification of BAL fluid in subjects, it was shown that the progression of COPD is associated with an increase in the number of non-polarized macrophages with poor phagocytic function (18). The phagocytic defects of alveolar macrophages are related to the sphingosine-1-phosphate (S1P) system, particularly the sphingosine-1-phosphate receptor 5 (S1PR5). Compared to healthy non-smokers/ex-smokers, the expression of S1PR5 mRNA in macrophages from COPD patients was significantly increased by more than 14-fold. By examining the DNA methylation levels of CpG islands in the S1P pathway genes, it was found that the methylation levels at these sites in macrophages from COPD patients were significantly lower compared to the control group, with COPD at 8.60 ± 2.53% and the control group at 27.85 ± 5.48% (78).Assessment of circulating monocytes from healthy non-smokers, smokers, and COPD patients revealed that the expression of EP300, HDAC2, and HDAC3 in peripheral blood monocytes from COPD patients was significantly reduced, while HDAC4 expression was increased. The reduction in HDAC2 may lead to chronic inflammation, airway remodeling, and obstruction. When monocytes from COPD patients were induced *in vitro*, they exhibited a differentiation trend toward the M2 type (CD163^+^ and CD206^+^) (79). Smoking causes genome-wide methylation changes in alveolar macrophages from healthy smokers and non-smokers, affecting 5mC and 5hmC, thereby altering gene expression and the affinity of transcription factors. The oxidative stress (ROS) induced by smoking leads to DNA damage and cytotoxic events and affects the formation of 5hmC through the TET enzyme-catalyzed DNA demethylation system. Differentially methylated genes associated with smoking include AHRR and CYP1B1, which are involved in the aryl hydrocarbon receptor pathway and metabolic activation. Transcriptional changes induced by smoking involve inflammatory responses, cancer, cell movement, migration, and adhesion processes, and may affect lipid metabolism and inflammatory responses through the LXR/RXR pathway, which has been validated in BAL cells (73).

### 3.4 Epigenetic regulation of macrophage development state

Gene expression is regulated by the structure of chromatin, where active gene regions typically show an open conformation, while regions of repressed genes are in a closed state (80). In the immune system, many mature blood cells derive from hematopoietic stem cells. These cells possess a highly plastic chromatin structure with a potential of cell differentiation (81). When cells differentiate into specific lineages, their chromatin structure becomes more stable. Genes associated with non-target lineages switch to a closed state. This stabilization of chromatin helps maintain specific patterns of gene expression. It ensures cell differentiation and maturation along defined pathways, thus preserving the specificity of cell function and the accuracy of genetic information transmission (82).

DNMT3A and TET2 are key epigenetic regulatory enzymes that play significant roles in the development and function of macrophages (83). By regulating DNA methylation, DNMT3A and TET2 influence histone modifications and chromatin structures. This subsequently affects the binding of transcription factors such as AP-1, RFX1 and KLF4. These transcription factors open chromatin to promote gene expression (84). TET enzymes such as TET2 catalyze DNA demethylation. TET2 particularly affects expression of genes associated with the actin cytoskeleton and phagocytic functions. These genes are important for the structure and behavior of macrophages including actin reorganization and cellular motility. DNMT3A and TET2 also regulate signaling pathways including ERBB2, PDGFRβ, CXCR4 and PIK3 (83).

In M1 macrophages, GDF15 mediates phosphorylation of ERBB2, leading to the stabilization of ligand binding and enhancement of downstream signaling through PI3K/AKT and MAPK/ErK pathways. These pathways regulate cytoskeleton arrangement, growth and stabilization of microtubules, which are essential for cell migration, podia formation and phagocytosis (85). PDGFR-β promotes actin cytoskeleton rearrangement, membrane ruffling and podia formation. Intracerebral hemorrhage (ICH) increased PDGFR-β expression in macrophages which induces their recruitment and the infiltration of inflammatory cells such as neutrophils (86). In immune cells, Fc receptors for IgG (FcγRs) are expressed and activated in monocyte-derived dendritic cells and macrophages. Binding of IgG-coated bacteria, viruses or other antigens to Fcγ receptors promotes clustering and tyrosine phosphorylation of Fcγ receptors by Src family kinases, which recruits tyrosine kinase Syk and PI3K, crucial for pseudopodia formation. Fcγ receptors also induce cell signal transduction mediated by ITAM or ITIM, and affect core functions of macrophages and dendritic cells, such as functional polarization, pathogen-killing ability and modulation of T cell responses. The immune system sets a strict threshold for phagocyte activation by activating Fcγ receptors and suppressing FcγRIIB expression (87). CXCR4, a member of the G protein-coupled receptor superfamily and an α chemokine receptor (88), activates multiple downstream pathways through CXCL12/CXCR4. This leads to increased cell proliferation and migration involving mediation by NF-κB (89) and PI3K/Akt pathways (90). CXCL12/CXCR4 also impacts the MAPK pathway through G proteins and regulates downstream molecules such as ERK1/2 and FAK, which facilitates chemotaxis and accumulation of inflammatory cytokines. CXCR4 plays a critical role in LPS induced acute lung injury. CXCR4 blockage can inhibit the production of inflammatory cytokines in macrophages by activation of MAPK and NF-κB signaling pathways (91). Macrophage migration inhibitory factor (MIF) is an inflammatory cytokine that activates the type II receptor CD74 and exhibits chemokine-like activity through interactions with chemokine receptors CXCR2 and CXCR4. Activation of the MIF-CXCR2 and CXCR4 axis promotes leukocyte recruitment, which can exacerbate atherosclerosis mediated by MIF (92).

### 3.5 Epigenetic regulation of macrophage physiological state

The microenvironment of the airway lumen significantly impacts the phenotype, function and regeneration of lung macrophages (93). Alveolar macrophages, a specific type of tissue-resident macrophage, primarily repopulate through *in situ* random cell division. This repopulation relies on M-CSF and GM-CSF, particularly after depletion from abnormal microenvironmental stimuli. During bone marrow transplantation, resident macrophages can maintain their proliferation capability in case the development of transplanted macrophages is impaired (94). In the lungs, alveolar macrophages persistently adapt to environmental changes. Environmental factors such as GM-CSF produced by respiratory epithelium can modulate chromatin structure of transplanted bone marrow precursors and reprogram differentiated macrophages. Lung macrophage-specific enhancers are enriched in regions involved in lipid and lipoprotein metabolism pathways. This indicates that lung macrophages play an important role in the metabolism of surfactant lipoproteins (93).

In long-term culture of mouse AMs, extensive epigenetic and transcriptional changes occur. These changes influence gene expression, signaling pathways and chromatin accessibility related to cell adhesion, migration, proliferation and immune responses as cells adapt to the culture environment. However, when these cells are reintroduced into their natural alveolar niche *in vivo*, epigenetic changes can be reversible. This reversibility is partially due to differences in open chromatin regions (OCRs), which are enriched with binding sites for core myeloid transcription factors such as PU.1, C/EBP and RUNX. These factors establish a core enhancer platform that determines macrophage identity and can converge additional signals-induced transcription factors. Key OCRs and the expression of genes such as AM-specific transcription factors (such as Car4 and Pparg) and surface markers (such as CD11c and SiglecF) are maintained during AMs cultures. This indicates that alveolar macrophages maintain a stable AM-specific identity during cultures. AMs also exhibit a wide range of responsiveness to environmental cues and adapt their gene expression *in vivo* and *in vitro*. This is consistent with the high plasticity of macrophages in response to diverse stimuli (95).

Lactic acid is a critical type of the microenvironment and serves as an important modulator of the immune system. In tissue microenvironments such as tumors and intestines, high concentrations of lactic acid exert immunosuppressive effects on both innate and adaptive immune cells (96). In tumor or inflammatory environments, high concentrations of lactic acid alter the metabolic state of macrophages and regulate their function and behaviors by epigenetic mechanisms. Lactic acid shifts macrophage metabolism towards the anti-inflammatory M2 phenotype (97). Cells detect the acidic pH environment through G Protein-Coupled Receptors (GPCRs) and influence M2 gene expression via the cAMP signaling pathway (98). As a fuel source for the TCA cycle, lactic acid promotes citrate accumulation. Lactic acid also affects histone acetyltransferase activity, such as p300, and thereby regulates the acetylation status of histone H3K27. Increased H3K27 acetylation levels are associated with the activation of anti-inflammatory genes and the suppression of pro-inflammatory genes. Long-term exposure of macrophages to lactic acid leads to their enduring immune suppressive functions. This state is attributed to long-term epigenetic reprogramming, akin to trained immunity. Lactic acid up-regulates genes associated with tolerance, such as Nr4a1, which helps macrophages adapt to the post-inflammatory recovery phase and thus maintain tissue homeostasis (99).

## 4 Epigenetic alterations of macrophages in COPD

### 4.1 DNA methylation of macrophages in COPD

Macrophage polarization plays an important role in COPD development (77). A potential mechanism for changes in gene expression of alveolar macrophages in smokers is the alteration of cytosine methylation in DNA coding sequences. In alveolar macrophages, differences in DNA methylation between heavy smokers and nonsmokers primarily target immune and inflammatory pathways (100). In COPD patients, epigenetic regulation controls phagocytosis of apoptotic cells by alveolar macrophages. Increased numbers of apoptotic epithelial cells in the airways necessitate their effective clearance by macrophages to prevent secondary necrosis and reduce inflammation (101). Overall levels of DNA methylation in alveolar macrophages from COPD patients are lower than those of healthy nonsmokers and smoking quitters, which may lead to deficiencies in phagocytic ability of alveolar macrophages in COPD patients (102). The sphingosine signaling pathway is essential for macrophage maturation and phagocytic function. In COPD patients, elevated mRNA levels of S1PR5 in macrophages are associated with lower levels of DNA methylation at CpG islands of S1PR5 compared to healthy nonsmokers and smoking quitters, suggesting that reduced methylation may account for the increased gene expression. DNA methyltransferase inhibitor 5-azacytidine treatment has been shown to rescue phagocytic defects in THP-1 macrophages caused by CSE in a dose-dependent manner (78).

Epigenetic alterations in macrophages lead to functional dysregulation and exacerbate lung tissue damage. Macrophages constitute approximately 90% of the cells in bronchoalveolar lavage (BAL) fluid. A genome-wide methylation analysis of BAL cells from COPD patients identified 1,155 differential methylation positions (DMPs) associated with COPD. However, functional analysis indicates that there is no significant correlation between DNA methylation and accelerated aging in COPD. Among these DMPs, 39% of them are located near COPD-related SNP sites. The DNA sequences or interacting DNA binding factors at these sites collectively determine DNA methylation patterns in these regions. Of the ten genes annotated with the most significant DMPs, four are associated with COPD severity, including NLRP3, SOX30, POMC and ZNF322 (103). NLRP3, encoding a cytoplasmic pattern recognition receptor, is a critical regulator of the inflammatory response as part of the NLRP3 inflammasome. Studies in both humans and mouse models have demonstrated a pivotal role for the NLRP3 inflammasome in the pathogenesis of COPD (104). Changes in expression of the transcription factor SOX30 are associated with the progression of emphysema in COPD, and are also correlated with the expression of Lsm10, a Sm-like protein associated with U7 snRNA. Lsm10 has previously been found to be involved in chronic mucus hypersecretion in COPD (105).

In some of chronic pulmonary diseases, such as COPD, asthma and cystic fibrosis, mucus obstruction is a significant manifestation in the airways. Mucus hydration refers to maintaining the proper moisture level in mucus to ensure its normal viscosity and flowability. The hydration state of mucus is crucial for its viscosity and mucociliary clearance (106). Scnn1b encodes the beta subunit of the epithelial Na channel in airway Clara cells. Scnn1b-Tg mice exhibit airway surface liquid (ASL) depletion, leading to mucus dehydration, reduced mucociliary clearance, and pronounced pulmonary disease characterized by airway mucus obstruction, inflammation, and spontaneous bacterial infections (107). The epigenetic landscape of AMs from Scnn1b-Tg mice includes pro-inflammatory changes. Studies show that in mucus-obstructive pulmonary diseases, these macrophages exhibit reduced DNA methylation and increased chromatin accessibility. These alterations mainly occur in promoter and enhancer regions, enabling the binding of transcription factors such as NFKB, AP-1, IRF2/3, CEBP, STAT6 and ATF3, which are associated with macrophage inflammation and polarization (108). After LPS stimulation, AMs in Scnn1b-Tg mice show impaired function, linked to increased activity of the IRF family of transcription factors. These factors are crucial in controlling macrophage polarization, inflammatory responses and remodeling (109). Increased activity of these factors may worsen the morbidity and mortality of COPD and cystic fibrosis (CF) patients during acute pulmonary exacerbations (110). Meanwhile, wild-type (WT) AMs under similar mucosal stimuli also display transcriptional programs akin to Scnn1b-Tg AMs and exhibit suppressed abilities of phagocytose and apoptotic cell clearance. This suggests that the pulmonary microenvironment can influence macrophage function and state through epigenetic mechanisms in a pathological context dependent manner. The transcriptional profile of Scnn1b-Tg AMs shows significant up-regulation of markers associated with both classical and alternative activation of macrophages, supporting the view that macrophage phenotype is a continuum, reflecting cellular adaptation to environmental disturbances (108). Kcnn4 encodes the calcium-activated potassium channel KCa.3.1, which is involved in ion secretion. Studies have shown that its activation increases the absorption of Na in airway epithelia, suggesting that KCa.3.1-induced hyperpolarization is sufficient to drive Na absorption. Silencing Kcnn4 in Scnn1b-Tg mice reduces Na absorption, decreases mucus adhesion in mice, and enhances mucociliary clearance efficiency. This suggests that alleviating the excessive Na absorption caused by the Scnn1b gene could be a strategy for treating mucus obstruction in COPD (111).

In COPD patients, alveolar macrophages and epithelial cells show increased methylation of α-Klotho mediated by Notch signaling. This is associated with elevated levels of DNMTs to reduce Klotho expression (112). The Klotho gene, identified as an aging-suppressant gene, is highly conserved in humans, rats and mice. Mice lacking Klotho exhibit aging symptoms such as growth arrest, skin atrophy, shortened lifespan and emphysema (113). Consistently, transgenic overexpression of Klotho extends lifespan, indicating its anti-aging and tumor-suppressive function (114). Exposure to CSE leads to reduced Klotho expression in MH-S and 16HBE cells. This reduction is associated with activation of the Notch signaling pathway and increased DNA methylation of the Klotho promoter. These changes by CSE are crucial for promoting the production of inflammatory cytokines and cell apoptosis. Specifically, inhibition of Klotho is associated with increased inflammatory responses and oxidative stress, involving pathways such as NF-κB, MAPK and Nrf2. This exacerbates the progression of COPD by promoting inflammation and apoptosis (115).

### 4.2 Histone acetylation of macrophages in COPD

Multiple mechanisms are involved in regulation of inflammation in COPD development. Inflammatory signal transduction in cells can be regulated by post-translational modifications (PTMs). The most common PTM is acetylation, where acetyl groups are added to lysine residues by histone acetyltransferases (HATs) or removed by histone deacetylases (HDACs). Current studies on epigenetic regulation of macrophage polarization focus on post-translational modifications of histones including histone methylation and acetylation, which are important for the polarization of M1 and M2 macrophages (116). Among HATs, E1A-binding protein p300 (EP300) and its homolog CBP can acetylate several proteins involved in regulation of lung function and glandular secretion (117, 118). EP300 plays a crucial role in the co-activation of various transcription factors including NF-κB and Activator Protein-1 (AP-1). Cigarette smoke induces abnormal and persistent lung inflammation. During smoke exposure, the acetylation of histones (H3/H4) and NF-κB by CBP/EP300 leads to increased levels of pro-inflammatory cytokines (119).

In peripheral blood mononuclear cells from patients with severe COPD, HDAC2 expression levels are significantly decreased. This decrease is closely associated with chronic inflammation, airway remodeling and obstruction (120). Rhinovirus infection in COPD patients leads to a significant increase in airway inflammation and expression of markers of oxidative and nitrosative stress. During acute COPD exacerbations, HDAC2 activity in macrophages decreases, negatively correlated with viral load, and marker levels of inflammation and nitrosative stress (116). HDAC3, a positive regulator of NF-κB-mediated inflammation, inhibits IL-4 induced M2 polarization (121), suggesting HDAC3 inhibition as a potential therapeutic approach for preventing inflammation in COPD (122). In IPF lung tissues, HDAC6 expression is increased. Tobramycin, a selective HDAC6 inhibitor, improves bleomycin-induced pulmonary fibrosis in mice by regulation of the TGFβ-PI3K-Akt pathway (123). A study of 105 COPD patients, including 42 smokers and 73 non-smokers, shows that HDAC2 expression is inhibited in circulating blood cells of COPD patients with mild, moderate, and severe conditions. This study also finds decreased HDAC3 levels in moderate to severe cases, increased HDAC4 expression in both mild and severe cases, and reduced EP300 levels in severe cases. Notably, studies show that a reduction in HDAC2 levels may lead to DNA damage and promote premature aging (79).

S1P is a critical regulator of cell fate and immune responses within the sphingolipid pathway (124). S1P is involved in immunological, cardiovascular and neurological processes via S1PR1 (125), and positively correlates with the release of pro-inflammatory cytokines by regulation of S1PR1 (126). S1P specifically interacts with HDAC1 and HDAC2, which are direct intracellular targets of S1P. This interaction functions to inhibit the removal of acetyl groups from lysine residues in histone tails, thereby reducing their enzymatic activity (127). In COPD patients, increased S1PR1 expression inhibits HDAC1 expression. Activation of the S1PR1/HDAC1 signaling pathway can cause macrophages to polarize towards an IL-12-positive M1 phenotype (128).

Particulate matter (PM) is also a major risk factor for COPD development. PM refers to a mixture of solid particles and aerosols in the atmosphere. Based on particle size and health impacts, it is categorized into PM_10_ and PM_2.5_ (129). Due to smaller size, longer atmospheric lifetime and greater health risks, PM_2.5_ is of more concern compared to PM_10_ (130). In PM-induced COPD mouse models, repeated exposure to PM leads to chronic inflammation and COPD development, which is primarily mediated by macrophages. PM triggers inflammatory responses in macrophages and advances COPD progression by modifying the epigenetic state of these cells, especially by altering histone acetylation levels of H4Kac. Specifically, PM exposure increases the chromatin binding of CTCF (CCCTC-binding factor) in macrophages. This affects the expression of enzymes in NAD^+^ synthesis pathways, such as KMO, KYNU, HAAO and QPRT, and inhibit NAD+ synthesis. These enzymes are crucial for intracellular NAD^+^ synthesis, essential for cellular energy generation and metabolic processes. In macrophages of PM-induced COPD mouse models, reduced NAD^+^ levels limit SIRT1 function (a NAD^+^ dependent histone deacetylase), leading to increased histone acetylation. This enhancement promotes the expression of inflammatory mediator genes, and exacerbates COPD process. SIRT1 inhibits the expression of inflammatory genes by deacetylating key transcription factors such as NF-κB and AP-1, and play a role in anti-inflammation. Therefore, activation SIRT1 or restoration of NAD^+^ metabolism might be effective strategies for COPD treatment (131).

### 4.3 Histone methylation of macrophages in COPD

Protein arginine methyltransferases (PRMTs) participate in the regulation of cellular processes such as transcription, RNA processing, signaling cascades, DNA damage response and liquid-liquid phase separation by methylating arginine residues (132). During COPD development, an immune process involves the extravasation of monocytes to the lung tissues, which is facilitated by PTMs (133). A study demonstrates that, in macrophages of COPD patients, expression of the epigenetic factor protein arginine methyltransferase 7 (PRMT7) is increased. Furthermore, the same study finds that reduced PRMT7 expression correlates with decreased monocyte recruitment to injury sites in a mouse model of COPD, which potentially alleviates COPD symptoms (134). The RAP1A gene regulates the adhesion and migration of monocytes (135). In monocytes, RelA-mediated activation of NF-κB can increase PRMT7 transcription and cause monomethylation of histone H3R2me1 at RAP1A regulatory elements, which enhances the control of inflammatory pathways. The continuous transformation of monocytes into macrophages, accompanied by their excessive accumulation, increases ALOX5 gene expression and the accumulation of its metabolite LTB4, which in turn triggers ACSL4 expression in lung epithelial cells, and ultimately causes extensive tissue damage. This process reflects the central role of monocyte-to-macrophage transformation and associated signaling pathways in regulating macrophage inflammatory responses (134).

PRMT6 also plays a protective role in CSE-induced emphysema. In a mouse model of CSE-induced emphysema, a lentivirus-mediated overexpression of PRMT6 can improve lung function. Overexpression of PRMT6 may inhibit the activation of the NF-κB/p65 pathway, and subsequently block the expression and release of pro-inflammatory cytokines (such as TNF-α and IL-1β) in the body, suggesting that PRMT6 acts as an anti-inflammatory agent (136).

### 4.4 Histone lactylation of macrophages in COPD

Lactate is an end product of glycolysis, which serves as a key energy source for mitochondrial respiration and also functions as a precursor for gluconeogenesis and a signaling molecule. Lactate-mediated reprogramming of immune cells and the enhancement of their cellular plasticity are crucial for establishing specific immune states associated with diseases (137). In glucose metabolism, there is a significant correlation between the rate of glycolysis and histone acetylation (138). In the tumor microenvironment and inflammatory diseases, lactate accumulation is common, where it acts as a key regulator of metabolism, immune responses and intercellular communication (139).

Additionally, lactate can link macrophage metabolic changes to epigenetic regulation. In tumor infiltration’s immunosuppressive environment, IL-4 drives macrophage polarization from M0 to M2 through glucose or TCA cycle metabolism. This process enhances histone acetylation, M2 gene transcription and functional immunosuppression (140). Inhaled PM2.5 particles can adhere to alveolar epithelial cells, and are engulfed by macrophages, causing oxidative stress and inflammatory responses (141). Experiments using C57BL/6J mice and RAW264.7 macrophages show that PM2.5 exposure significantly increases lactate dehydrogenase activity and its levels in macrophages, thereby promoting glycolysis. This shift in metabolism results in increased histone lactylation in macrophages. PM2.5-induced glycolysis enhances histone lactylation in promoters of pro-fibrotic genes such as PDGF, ARG1, VEGFA and THBS1, and significantly increases lactylation at these sites. Activating TGF-β/Smad2/3 and VEGFA/MEK/ERK pathways promotes the epithelial-to-mesenchymal transition (EMT) of lung epithelial cells. The LDHA inhibitor GNE-140 effectively alleviates PM2.5-induced lung inflammation and fibrosis by inhibition of glycolysis and subsequent histone lactylation in mice (142). Furthermore, the acetyltransferase p300 is involved in the lactylation of histones (143). Knockdown of p300 in macrophages significantly reduces lactate-induced histone lactylation, which decreases the expression of pro-fibrotic genes (144).

### 4.5 miRNA expression of macrophages in COPD

MicroRNAs (miRNAs) are endogenous non-coding RNAs with 21-24 nucleotides long, which regulate gene expression post-transcriptionally by binding to imperfectly complementary sites on target mRNAs to inhibit protein translation (145). In COPD patients and smokers, miRNA-486-5p expression has been shown to be upregulated in alveolar macrophages and peripheral monocytes with a positive correlation with levels of IL-6, IL-8, TNF-α and IFN-γ. Increased expression of miR-486-5p suppresses expression of HAT1 by targeting its 3’ UTR, subsequently enhancing the inflammatory response triggered by TLR4 (146). miR-149 expression is reduced in whole blood in smokers compared to those of non-smokers. CSE stimulation inhibits miR-149 expression and increases TLR-4 levels in THP-1 cells. miR-149 inhibitor treatment upregulates TLR-4, while miR-149 overexpression reverses CSE effects in THP-1 cells. Changes in miR-149 expression lead to alterations in TLR-4 and NF-kB p65 protein levels, thereby regulating inflammation (147).

RAW264.7 cells stimulated with CSE show an increase in miR-21 expression, which continues to rise with prolonged CSE exposure. In wild-type mice used as a COPD model, miR-21 expression in bronchial-derived macrophage-derived dendritic cells (BDMDs) is significantly elevated. Knockout of miR-21 in mice undergoing a COPD modeling procedure results in significantly reduced alveolar damage compared to wild-type mice. The ratio of M2 to M1 macrophages correlates positively with miR-21 expression. An miR-21 inhibitor can reduce expression of inflammatory factors associated with M2 macrophages in RAW264.7 cells stimulated with CSE (148).

In COPD patients, miR-223 expression in lung tissues are higher than in healthy controls. miR-223 influences the function of neutrophils, and higher miR-223 expression may alleviate pulmonary inflammation in mice by regulating the activity of the NF-kB pathway, demonstrating its anti-inflammatory effects. Depletion of miR-223 leads to enhanced inflammatory responses (149).

The long non-coding RNA (lncRNA) miR155HG also serves as a regulator of macrophage polarization and is highly expressed in COPD patient macrophages induced by GM-CSF. There are NF-kB binding sites upstream of the miR155HG transcription start site, whose binding upregulates the expression of the miR155HG promoter. miR155HG participates in macrophage polarization by regulating the expression of TNF-α, IL-1β, IL-10 and IL-12. Overexpression of miR155HG promotes M1 polarization and the release of pro-inflammatory cytokines in GM-CSF-induced macrophages. Knockdown of miR155HG inhibits M1 macrophage polarization and enhances M2 macrophage polarization (150). In miR155 knockout mice, the key COPD related genes MMP12 and ADAM19 expression in alveolar macrophages is reduced, which can prevent elastase induced emphysema and dysfunctional changes (151).

Let-7 miRNA was first discovered in Caenorhabditis elegans and is highly conserved in human tissues, comprising 12 family members. Let-7 can target the mRNA expression of many genes, regulating cellular proliferation, differentiation, metabolism and apoptosis (152). Let-7 influences macrophage polarization in COPD. Let-7 expression in the whole blood, serum, BAF cells and lung tissues of COPD patients are significantly lower than in healthy controls. In a co-culture system of HBE and THP-1 derived macrophages stimulated with 5% CSE, mRNA expression of M1 polarization genes (iNOS and TNF-α) increases from 0-24 hours and decreases at 48 hours. M2 polarization genes (Arg1 and TGF-β) show little change within 24 hours but significantly increase at 48 hours. This indicates that with the prolonged exposure to smoke, bronchial epithelial cells induce a shift in macrophage polarization from M1 to M2. In COPD patients and COPD mouse models, the number of lung macrophages increases, with a greater proportion of M2 cells compared to M1 cells. MMP9/12 expression increase released by M2 macrophages. Smoke exposure can downregulate Let-7 expression in HBE cells and induce the formation of M2 macrophages. In the inflammatory pathway, the IL-6/STAT3 pathway promotes the polarization of M2 macrophages (153). Let-7 can reduce M2 polarization in alveolar macrophages by inhibiting the IL-6/STAT3 pathway, thereby decreasing MMP release and slowing the progression of severe emphysema (154).

## 5 Conclusion and future perspectives

### 5.1 Summary

**Figure 3:**
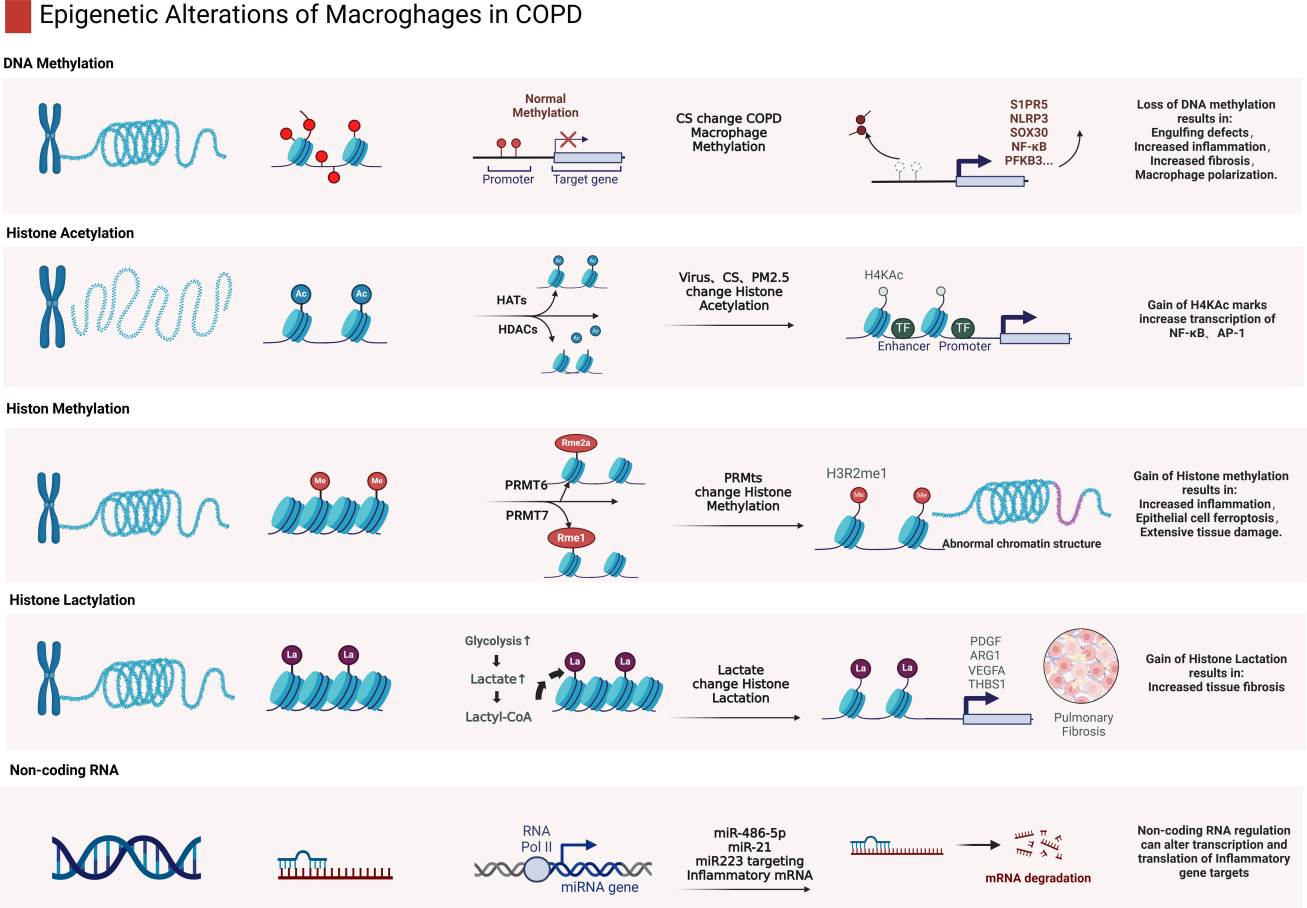
Epigenetic alterations of macrophages in COPD. This figure provides a detailed depiction of various epigenetic modifications in macrophages in COPD conditions, involving DNA methylation, histone acetylation, methylation, lactylation and impacts of non-coding RNAs. DNA methylation changes often lead to decreased phagocytic functions, such as impaired clearance of apoptotic cells. Histone acetylation is generally associated with upregulation of pro-inflammatory factors, increased transcriptional activities of NF-κB and AP-1. Alterations in histone methylation can lead to abnormal chromatin structures, affecting macrophage function. Histone lactylation is associated with the progression of pulmonary fibrosis. Non-coding RNAs, such as miRNAs and long non-coding RNAs, can mediate macrophage polarization and function by regulation of expression of inflammation-related genes. (Created with BioRender.com).

Epigenetic factors regulate macrophage polarization by mediating DNA methylation (78, 100, 103) and histone modifications such as acetylation and lactylation, which mediates the transition of reparative M2 to inflammatory M1 phenotypes (128, 148) and the expression of pro-inflammatory and anti-inflammatory genes in macrophages (**Figure 3**) (29, 79). In COPD, changes in epigenetic modifications such as DNA methylation and histone acetylation not only reduce the phagocytic capabilities of macrophages but also enhance inflammatory responses by modulating the activity of genes involved in inflammation and immune regulation (119).

Epigenetic factors such as PRMTs (134, 136), HDACs (116, 131) and HATs (117) affect several key signaling pathways including NF-κB, STAT and PI3K/Akt (30, 46, 85), which is associated with or contributes to COPD development. These factors mediate macrophage function and polarization by regulating the methylation and acetylation states of critical proteins in these signaling pathways.

Furthermore, epigenetic modifications such as histone acetylation and methylation alter chromatin structure and affect the binding of transcription factors to gene promoters. These effects alter expression of genes related to inflammation and immune responses in macrophages (131). These changes are associated with the clinical manifestations of COPD. Specifically, epigenetic modifications modulate macrophage-mediated inflammatory responses and tissue repair, which influences lung function and acute exacerbations in COPD.

### 5.2 Future perspectives

Understanding epigenetic regulation in COPD opens promising avenues for developing targeted therapies. Several potential therapeutic approaches and research directions are as follows:

Epigenetic Modifiers: Given the roles of DNA methylation and histone modification in COPD, drugs which can modify these epigenetic regulations might serve as effective therapeutic candidates. For example, inhibitors of DNMTs and HDACs could be used to reprogram macrophage functions towards a less inflammatory state. In sepsis-induced inflammatory diseases such as acute lung injury (ALI), treatments with DNA methyltransferase inhibitors such as 5-Aza-2’-deoxycytidine (Aza) and histone deacetylase inhibitors such as Trichostatin A (TSA), as well as a combination (Aza +TSA), have been shown to significantly attenuate lung tissue pathological changes and inflammation in LPS-induced mice. Aza +TSA treatment of LPS-stimulated macrophages significantly reduces levels of pro-inflammatory factors due to dual inhibition of the MAPK-HuR-TNF and STAT3-Bcl2 pathways, and reduces ALI inflammation and promotes an anti-inflammatory M2 macrophage phenotype (155).

RNA-based Therapies: Targeting m6A RNA methyltransferases such as METTL3 and FTO could regulate macrophage activation and polarization, and mediate inflammation in COPD. Small molecule inhibitors or RNA-based strategies could be employed to modulate the activity of these enzymes. Non-coding RNAs (ncRNAs) are an essential group of RNAs within the transcriptome, composed of various endogenous RNA molecules. Different ncRNAs are also candidates in gene expression regulation associated with M1/M2 polarization. For example, an M1 predominance over M2 is a major pathological feature in Multiple Sclerosis (MS). The long non-coding RNA (lncRNA) GAS5 has been identified as an epigenetic regulator of microglial polarization. GAS5 inhibits the transcription of TRF4, a key factor controlling M2 macrophage polarization, by recruiting the Polycomb Repressive Complex 2 (PRC2), and modulates M2 polarization (156).

Gene Therapy: Advances in gene editing technologies such CRISPR/Cas9 allow the development of specific genetic interventions to correct or modify gene expression in macrophages. Such approaches could directly target pathways involved in macrophage dysfunction and chronic inflammation. For example, activation of HIF1α in tumor-associated macrophages can be epigenetically silenced by modulation of histone H3 methylation in its promoter regions using the CRISPR/dCas9-EZH2 system. The histone H3 methyltransferase EZH2 can be specifically recruited to the promoter area. Silencing Hif1α in macrophages results in a heritable tumor-suppressive phenotype, demonstrating the potential of epigenetic reprogramming to alter macrophage phenotypes (157).

Biomarker Development: The clinical use of biomarkers for the treatment of COPD has also been a significant research direction in recent years. Recently, Sanofi and Regeneron Pharmaceuticals’ major drug, dupilumab (brand name: Dupixent), was approved by the The European Medicines Agency (EMA) for marketing as an add-on maintenance treatment for patients with uncontrolled COPD (Meeting highlights from the Committee for Medicinal Products for Human Use (CHMP) 27-30 May 2024. Retrieved July 18, 2024, from https://www.ema.europa.eu/en/news/meeting-highlights-committee-medicinal-products-human-use-chmp-27-30-may-2024#positive-recommendations-on-extensions-of-indications-67242). It is noteworthy that the EMA is the first global regulatory body to approve Dupixent for COPD patients, with regulatory authorities in the United States, China, and Japan currently reviewing the submitted research materials. This approval is based on the results of the Phase III BOREAS and NOTUS trials, which demonstrated significant efficacy of dupilumab in reducing acute exacerbations and improving lung function(Dirk Einecke,Ein Antikörper gegen schwere COPD, Pneumo News, 16, 3 (58–58), (2024). https://doi.org/10.1007/s15033-024-4026-6).

In conclusion, the epigenetic mechanisms discovered in preclinical research combined with clinical data on COPD further support the importance of macrophage polarization in the progression of the disease. Further research into COPD-specific epigenetic changes could lead to the development of new biomarkers for early diagnosis, disease monitoring, and treatment evaluation (158, 159). This not only helps us understand the mechanisms of disease development but also provides potential targets for future therapeutic strategies, facilitating earlier interventions and potentially improving the prognosis of COPD (160).
